# Influential Factors on Diffusion Bonding Strength as Demonstrated by Bonded Multi-Layered Stainless Steel 316L and 430 Stack

**DOI:** 10.3390/ma17153713

**Published:** 2024-07-27

**Authors:** Da-Wei Liu, Chun-Nan Lin, Wei-Shuai Lin, Shyong Lee, Jyh Gwo

**Affiliations:** 1 Department of Mechanical Engineering, National Central University, Taoyuan City 32001, Taiwan; 2 National Chung Shan Institute of Science and Technology, Taoyuan City 32500, Taiwan

**Keywords:** diffusion bonding, multi-layered, SS316L, SS430, stainless steel

## Abstract

In this study, we optimized the parameters of diffusion bonding on multi-layered stainless steel 316L and 430 stacks. The preparation process for diffusion bonding is crucial, as the bonding surfaces need to be polished and meticulously cleaned to ensure a smooth bonding process. We fabricated twelve-layer plates consisting of 55 mm × 55 mm × 3 mm and 100 mm × 50 mm × 3 mm dimensions, and the bonding response was investigated by evaluating the tensile strength of the bonding zone under varying bonding conditions, with a bonding temperature ranging from 1000 to 1048 °C, a bond time ranging from 15 to 60 min, pressure ranging from 10 to 25.3 MPa, and under a vacuum environment. SS430 exhibits a significantly higher compression creep rate than SS316L. The compressibility of diffusion welding materials does not impact the diffusion bonding strength. Multi-axial tensile strength tests confirmed strong bonding joint strength in various axes. The tensile strengths of monolithic and Diffusion bonding (DB) specimens tested in parallel are essentially identical. The optimized diffusion bonding parameters (Condition G2C: 1048 °C/25.3 MPa/15 min) are ideal for producing SS316L stainless steel cores in compact heat exchangers, offering a superior bonding quality and reduced costs. These findings have practical implications for the production of stainless steel cores in compact heat exchangers, demonstrating the relevance and applicability of our research.

## 1. Introduction

Diffusion bonding (DB), explosive welding, and accumulative roll bonding (ARB) are three commonly used metal joining techniques, each with distinct advantages and disadvantages. DB offers high bonding strength comparable to the base material itself. It does not require filler materials, which reduces the potential for corrosion issues. Additionally, DB operates at lower processing temperatures, avoiding liquid phase formation and deleterious microstructural changes, thereby ensuring the integrity of the materials. However, this process can be time-consuming and costly due to the precise control of the conditions required. The surfaces to be joined must be meticulously cleaned and free of oxides to ensure high-quality bonds, which can complicate the preparation process [1]. Explosive welding, on the other hand, is a rapid process capable of achieving high-strength bonds in a very short time. It is particularly suitable for joining large metal plates, making it highly efficient for large-scale production. Furthermore, it can join dissimilar materials, including metals that are difficult to bond using other methods. However, explosive welding poses significant safety risks and requires stringent safety measures. Additionally, the process can alter the microstructure at the bonding interface, potentially affecting the material performance [2]. ARB is known for significantly enhancing the strength and hardness of materials. It is well-suited for large-scale and continuous production, offering high-production efficiency. The process maintains good ductility in the materials even after multiple ARB cycles. Nevertheless, the quality of the interface can sometimes be compromised, with potential micro-cracks or other defects requiring further processing and inspection. The process also demands specialized rolling equipment and high-pressure apparatus, leading to substantial initial equipment costs [3]. In summary, DB is ideal for applications requiring the precise control of bonding conditions and high-strength multi-layer structures. Explosive welding excels in the rapid joining of large metal sheets, while ARB is advantageous for large-scale continuous production and material strengthening. The choice of an appropriate joining technique depends on specific application requirements to achieve optimal results [4].

The increasing demands for efficiency, compactness, and safety in industrial applications, such as the next generation of high-temperature reactors, highlight the importance of advanced heat exchanger technology. These reactors, including the high-temperature gas-cooled reactor and the advanced high-temperature reactor (AHTR), aim to enhance energy efficiency in electricity production and provide high-temperature heat for industrial processes. Effective heat exchangers are crucial for transferring energy between the nuclear heat transport system and industrial process heat systems. However, achieving this efficiency requires overcoming challenges related to cost, size, reliability, robustness, maintenance, and longevity.

DB has shown promise in the fabrication of heat exchangers due to its ability to produce high-quality joints with minimal deformation. It is particularly relevant for high-temperature applications where materials such as stainless steel SS316L are commonly used. The effectiveness of DB depends on various parameters, including time, temperature, pressure, and surface preparation. For instance, chromium oxide layers, which form naturally on stainless steel, must be managed to ensure successful bonding. Techniques such as surface cleaning, nickel plating, or the use of nickel foils are often employed to facilitate bonding and enhance joint integrity.

In the context of microchannel heat exchangers, DB offers significant advantages. Microchannels provide a high surface area-to-volume ratio, enabling efficient heat transfer and compact designs. However, the complexity of microchannel geometries and the need for uniform bonding quality across multiple layers pose significant challenges. Ensuring a consistent pressure application and maintaining joint integrity throughout the stack of layers is critical for the reliability and performance of the heat exchanger.

Stainless steels such as SS316L and SS430 have good surface quality and strong corrosion resistance, and both are suitable for microchannel heat exchangers. However, the DB of stainless steel can be challenging due to the presence of a native surface oxide layer [5]. This oxide layer acts as a diffusion barrier, hindering the interdiffusion process between the bonding surfaces. This is because the oxide layer exhibits high stability and has minimal solubility in the parent metal, even under elevated temperatures [6]. To address these challenges, traditional DB processes typically require either/or a combination of bonding surface preparation techniques, chemical cleaning methods, and the use of a vacuum environment [7,8].

Some researchers have presented the joining of SS316 stainless steel by DB with and without an interlayer [9]. An et al. [10] investigated DB of SS316L–SS bars with and without Ni foil interlayers at 850–1050 °C, under 10 MPa pressure for 1 h. The study explored the relationship between the bonding parameters and tensile strength at elevated temperatures. The results suggested that the Ni interlayer reduces the room-temperature strength but enhances the high-temperature strength due to the transformation of Fe_0.64_Ni_0.36_ into FeNi_3_. Optimized processing parameters were recommended based on these findings. Harumoto et al. [11] systematically investigated the influence of cold rolling on diffusion bondability using SUS316L stainless steel sheets with reduction ratios of 0%, 35%, 50%, and 70%. The tensile testing results showed that highly rolled sheets exhibit enhanced diffusion bondability, attributed to changes in the microstructure. Li et al. [12] conducted microscopic tensile tests on 316LSS vacuum diffusion-bonded joints to study the microstructure evolution and the effect of micro-voids on interfacial failure. In situ observations showed cracks most likely initiate at grain boundaries, particularly those oriented at 0–20° to the loading axis. Intergranular cracks dominate interfacial failure and micro-voids only link up when the load reaches 352 MPa. The microstructure of the joints primarily determines interfacial failure. Mateus et al. [13] provided an overview of a study to determine suitable parameters for bonding AISI 316L stainless steel. Diffusion-bonded samples were used to machine 40 specimens for tensile tests and microstructural analysis. It was concluded that temperature most significantly influences bond quality by enhancing mass transport. The optimal temperature was found to be 1040 °C, yielding the best results regardless of pressure and time, which showed similar mechanical properties. These parameters will be used in fabricating SS316L stainless steel cores for compact heat exchangers.

Several studies have explored the fusion of ferritic stainless steel with various materials through DB, both with and without the incorporation of an interlayer [14,15,16]. However, no relevant literature exists regarding the joining of ferritic stainless steel by DB.

This research article focuses on the innovative use of multi-layer stacking in DB of stainless steel 316L and 430, which has not been given prior attention. The goal is to enhance the performance and reliability of heat exchangers in high-temperature industrial applications, addressing the need for advanced fabrication methods that can meet the stringent demands of modern energy systems.

Diffusion bonds with SS316L and SS430 were prepared, respectively, and the influence on the diffusion bondability of SS316L and SS430 stainless steel sheets was systematically investigated from the viewpoint of the microstructure. Examining the influence of the process parameters on the bonding strength of these materials contributes to the optimization of DB techniques for multi-layered structures. It will be noticed that perfect genuine DB can be obtained with a bond strength indistinguishable with respect to the parent monolithic material, while non-genuine DB with an interlayer cannot [17].

## 2. Experimental

The experimental process of DB research includes tensile testing and elongation measurement. The experimental planning of diffusion welding joint parameters includes (1) material, (2) number of bonding layers, (3) size of the bonding material, (4) bonding temperature, (5) bonding pressure of the test piece, (6) bonding time, (7) joint vacuum, and (8) surface treatment of the joint surface of the test piece before joining and measurement of dimensional changes before and after bonding.

### 2.1. Materials

In the present research, SS316L and SS430 stainless steel were used as the material for a multi-layered stack; their chemical compositions are shown in Table 1.

### 2.2. Pre-DB Preparation Methods

SS316L and SS430 stainless steel are selected as the target materials used in this DB study. The material of SS316L is designed into two groups of layered laminate contour: The first group, designated as G1, consists of twelve blocks and each pre-bond block is 100 mm × 50 mm × 3 mm. The second group, designated as G2, contains twelve layers, and each pre-bond piece is of 55 mm × 55 mm × 3 mm. The material of SS430 is designed as one group of layered laminate contours; each pre-bond block is 55 mm × 55 mm × 3 mm. The faying surfaces were polished with 0.1-μm alumina powder and ultrasonically cleaned in acetone. The specimens were rinsed with distilled water and dried with compressed air to prevent recontamination before the DB process.

### 2.3. DB Process and Conditions

**Figure 1:**
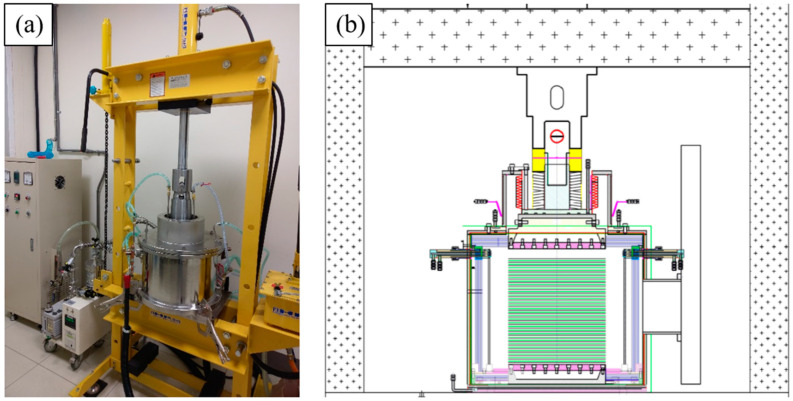
The experimental setup for DB: (**a**) Appearance of the machine. (**b**) Schematic of DB machine structure.

**Figure 2:**
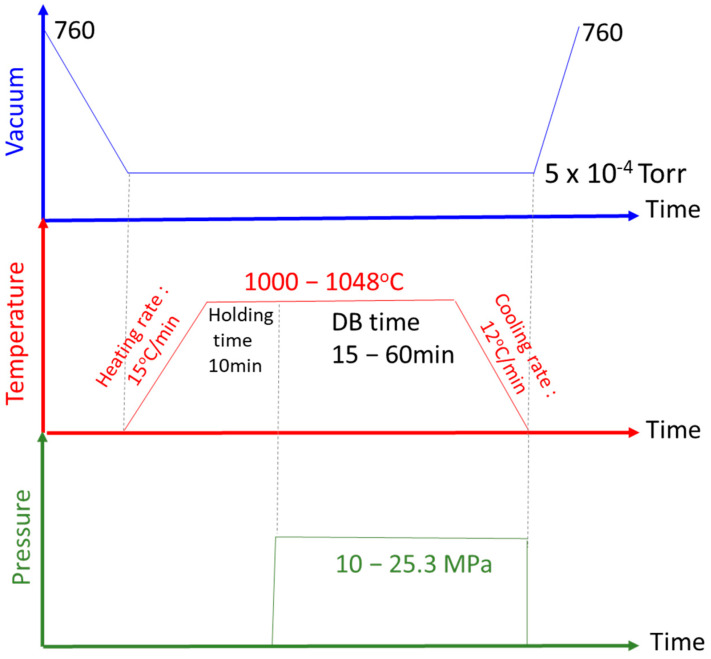
Schematic illustration of the DB process.

Figure 1 shows specimens for the twelve plates of each material stacked in a fixture and placed in a diffusion welding equipment vacuum chamber. First, the vacuum pump was turned on and the vacuum chamber was evacuated to 5 × 10^−4^ Torr. The chamber was heated to an experiment-set temperature with a heating rate of 15 °C/min. Once the target temperature was reached, the specimens were left for 10 min to stabilize the temperature. After that, the specimens were then thermally pressed, and the pressure and the DB time were according to that which was experimentally planned. During the DB period, we kept checking the pressure gauge; once the pressure was reduced, we needed to increase the pressure manually. If pressure was reduced continuously, the specimens would deform seriously, and the DB process would fail. To prevent the temperature of the vacuum chamber from becoming too high, a cooling water system was implemented. After the DB process was completed, the heating device was turned off. The cooling system within the vacuum chamber continued to operate, reducing the specimen’s temperature to room temperature at a rate of 12 °C/min. Finally, the pressure applied to the specimen was released, and the vacuum chamber was turned on. Figure 2 illustrates the temperature and pressure changes over time

Based on the conclusions from previous studies on DB parameters of SS316L [13], higher temperatures, more significant pressure, and longer durations all contribute to improving the quality of bonding. However, using extreme values for each parameter can cause severe deformation of the specimens, leading to DB failure. To balance the bonding quality and reduce DB costs, we referred to the results of prior research (as shown in Table 2) [12,13,18,19] to design the experimental conditions for this study, as shown in Table 3. Condition G1 is for demonstration of the larger-area SS316L DB capability. The condition G2 group has four branches, G2A, G2B, G2C, and G2D, which are used to optimize the SS316L DB process. The last condition is for SS430.

### 2.4. Test Pieces’ Preparation

**Figure 3:**
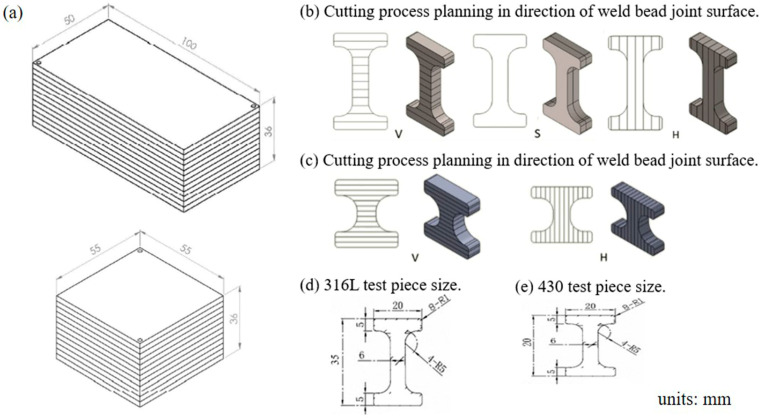
Schematic diagram of (**a**) the contour of two different dimensions; (**b**) the SS316L test piece in V, S, and H direction; (**c**) the SS430 test piece in V and H direction; (**d**) SS316L test piece size; (**e**) SS430 test piece size.

After the DB process, the G group of SS316L was a twelve-layered contour containing eleven DB interfaces with heights of ~35 mm (G1), ~35.10 mm (G2A), ~34.96 mm (G2B), ~34.76 mm (G2C), and ~35.30 mm (G2D), which was able to make tensile specimens, respectively. The SS430 was a twelve-layered contour containing eleven DB interfaces with a height of ~20.10 mm, which was also able to make tensile specimens. The tensile test of SS316L was planned with three types of specimens: V, S, and H, primarily based on the orientation of the DB surface for cutting. The tensile test for SS430 was planned with two types of specimens: V and H, primarily based on the orientation of the DB surface for cutting. The route of preparing test specimens is depicted in Figure 3.

## 3. Results

### 3.1. Impact Testing

**Figure 4:**
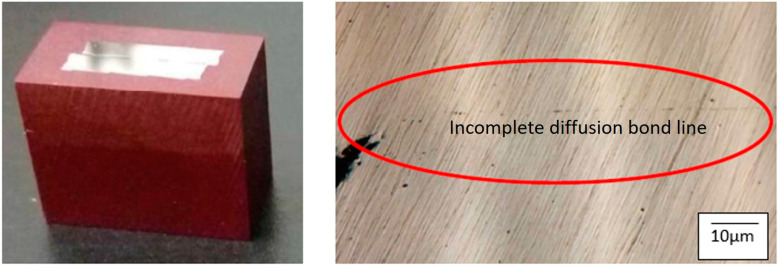
Optical micrographs of the specimen-bonded interface region with the G1 of SS316L after press bonding, the red circle is the incomplete diffusion bonding line.

**Figure 5:**
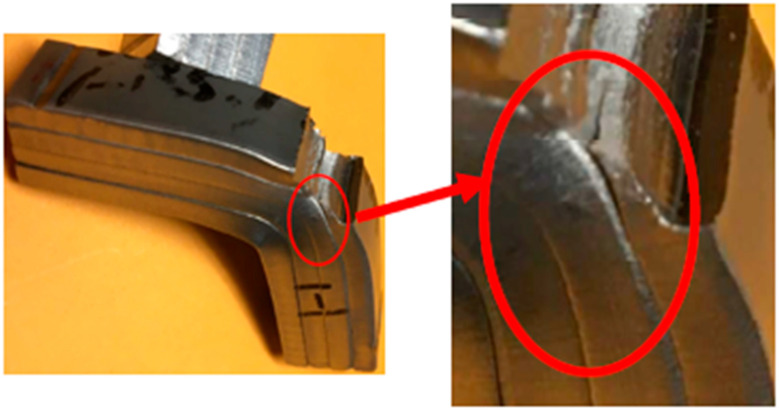
Appearance of a notched specimen experiencing severe bending at the region.

Using the G1 group of the SS316L material, a preliminary analysis revealed that there was not a complete diffusion bond between the two metals, as shown in Figure 4. A notched specimen was impact tested at room temperature, as shown in Figure 5, and the results indicated that delamination only occurred in the region experiencing severe bending.

### 3.2. Tensile Test

Tensile test planning involves three types of test pieces: V, S, and H, for SS316L test pieces, which are categorized into V, S, and H types, as shown in Figure 3b, and another for SS430 test pieces is divided into V and H types, as shown in Figure 3c. In these designations, V, S, and H represent the relationship between the weld bead and the stretching direction. The designation of the V test piece indicates that the weld bead is perpendicular to the stretching direction. The designation of the S test piece means the test piece is cut along the joint surface of the weld bead, and the weld bead is parallel to the tensile direction. The designation of the H test piece means that the test piece design is cut across the joint surface of the weld bead, and the weld bead is parallel to the tensile direction. For SS316L I-shaped tensile test pieces, the V, S, and H directions are divided into five groups of codes: G1, G2A, G2B, G2C, and G2D. There are two test pieces in each group, five groups of ten pieces in the V direction, five groups of ten pieces in the S direction, and five groups of ten test pieces in the H direction. Three tensile test pieces of the SS316L parent material were also made as a control group. The height of the SS316L test piece was ~35 mm and 20 mm in width, with a thickness of 5 mm in the V, S, and H directions. The size of the parent material test piece was the same as that of the V-, S-, and H-direction test pieces, see Figure 3d for details. For the SS430 I-shaped tensile test pieces, six test pieces in the V direction and six test pieces in the H direction, three tensile test pieces of the SS430 parent material were also made as a control group. The height of the SS430 test piece was ~20 mm and 20 mm in width, with a thickness of 5 mm in the V and H directions. The size of parent material test piece was the same as that of the V- and H-direction test pieces, see Figure 3e for details.

**Figure 6:**
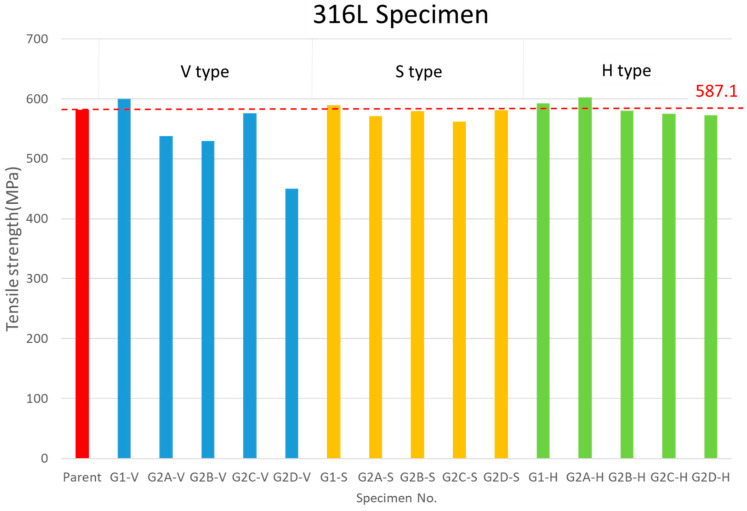
Ultimate tensile test results of the SS316L.

A tensile fixture was designed simultaneously to ensure that the applied force of the stretching machine was parallel to the test piece. The fixture was divided into two parts, both made of the same material as the test piece. The groove, with a depth of 11 mm, matched the size of the I-shaped tensile test piece. Table 4 and Figure 6 show the SS316L room-temperature DB tensile test results according to the V, S, and H directions, respectively. The raw material test piece number is Mono-SS316L-1~3. The characteristic of the H direction test specimen is that it has a plurality of DB joint surfaces parallel to the tensile direction, and the joint surface is located on the front of the specimen. The research observed that the strength after stretching is close to the monolithic material, and the necking constriction phenomenon of the specimen side is similar to the monolithic material. The result proves that a multi-layered joint is no different from a monolithic metal. Some test pieces were observed where the specimen was separated by DB joints, and they showed more than two necking constrictions. This can be further quantified by calculating the joint ratio of the test piece in the H direction, indicating that all multi-layered DB joints have well bonding. From the experimental results after the stretching of multi-layered joints, it is verified that DB is complete. The characteristic of the S direction test piece is that it has only a single DB joint surface parallel to the tensile direction. The joint surface is located on the side of the test piece and has a different cross-sectional area than the H-direction test piece. The necking constriction phenomenon of the test piece’s side is the same as that of the monolithic material. It has the same results as the H direction; the specimen is separated by the DB joint, showing more than two necking constrictions. In addition to verifying that the single-joint material is no different from the monolithic metal, it has also proven that bonding is good. It can be explained by calculating the DB ratio. The elongation of the test piece in the H and S directions is higher than the monolithic metal, and it does not differ due to different bonding joints.

The test piece in the V direction has delamination bonding, and the tensile direction is perpendicular to the DB joint surface. Compared with the tensile strength of the monolithic material, it is found that the tensile strength is generally lower than the base material, and the necking constriction of the test piece’s side is not obvious. There is even almost no necking, and the fracture cross-section is generally flat. After stretching each test piece, the fracture position of each piece is completely different, and the judgment surface is the DB surface. It is found from the experimental results that when the stretching direction is perpendicular to the DB multi-layered joint, it is difficult to predict the fracture point. Test pieces belonging to the V1 group were well bonded, but after the experiment, it was also found that the test pieces’ side still had no necking constriction. The multi-layer adhesive joint was perpendicular to the tensile direction and influences the joint and force direction of H and S specimens: (1) The DB ratio of the multi-layered joint perpendicular to the tensile direction, and tensile strength, can be found to be poorer than in H and S group specimens. When the direction of stretching is perpendicular to the joint surface, the bonding joint of the material itself can be regarded as in a poor condition. Therefore, in the future, we can further study the bonding ratio to determine how the angle relationship between the bonding surface and the tensile direction will affect strength. (2) When multiple layers are joined, due to a large number of bonding joints, it is difficult to predict where the material will break.

The elongation of the test piece in the V direction is obviously lower than the monolithic material due to there being no necking constriction. Through experiments, SS316L displays a compressibility of 1321 K, which is higher than that of 1273 K. There is no difference in a compressibility between 25.3 MPa and 20 MPa, and a compressibility of 10 MPa is the lowest. Taking bonding time as a parameter, the longer the bonding time is, the lower the compression rate will be. From the analysis, it can be found that a high bonding temperature, high bonding pressure, and short bonding time increase the impact on compressibility, where SS316L is 1.9%~3.4%, as listed in Table 4 for SS316L.

**Figure 7:**
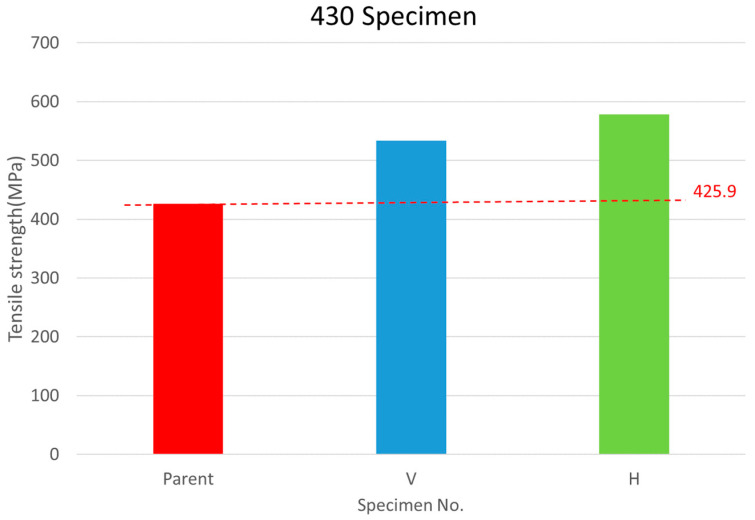
Ultimate tensile test results of the SS430.

The SS430 DB Tensile Test (refer to ASTM E8/E8M-22 [20]) results at room temperature are shown in Table 5 and Figure 7 according to the V direction and H direction, respectively. The extended codes V and H are given according to the design of the test piece. The base metal number is Mono-430-1~3. An experimental study found an angle relationship between the bonding joint and tensile force direction. When the tensile force direction is in the same direction (0°) as the bonding joint, no matter whether there is a single-layer or multi-layer joint, the necking of the test piece can be clearly observed for SS316L and SS430. The shrinkage phenomenon and bonding strength are generally higher than the strength perpendicular to the stretching direction (90°). The test results of SS316L can clearly indicate that these two phenomena can distinguish between the fracture of the test piece at the bonding joint surface and the fracture of the monolithic metal, while SS430 is not easy to distinguish. However, the tensile strength and material bonding ratio of SS430 are higher than those of the monolithic material. It is speculated that a high compression ratio makes SS430 DB ratios higher. When the stretching direction is perpendicular to the bonding joint (90°), no necking phenomenon of the test piece was observed for SS316L and SS430, and the test results of SS316L and SS430 can clearly indicate the phenomenon that the test piece breaks at the bonding joint surface.

The V-direction test piece elongation is the same as the SS316L test result, which is obviously lower than that of the monolithic material due to the absence of necking.

Through experiments, where SS430 was bonded at a high bonding temperature, high bonding pressure, and appropriate bonding time, it can be predicted that there will be a phenomenon of a high compression ratio. An experiment found that SS430 has a higher compression ratio than SS316L, up to 44%, as listed in Table 5 for SS430. This is because SS430 only contains 0.75 wt% nickel, so its high-temperature strength is much worse than that of SS316L which contains 12 wt% nickel (see Table 1).

### 3.3. Fracture Surface

**Figure 8:**
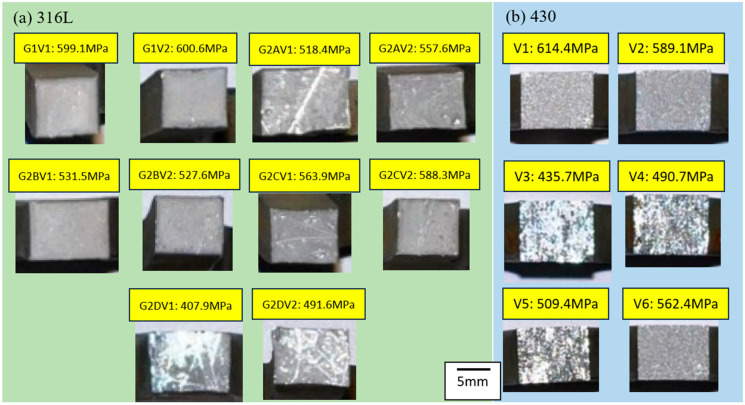
(**a**) The fracture surface of the SS316L specimens. (**b**) The fracture surface of the SS430 specimens.

During the macroscopic examination of the tensile specimens of SS316L and SS430 stainless steels, an interesting phenomenon was observed. Surfaces exhibiting higher tensile strength appeared darker and lacked glossiness, while those with lower strength displayed a brighter appearance. In this experiment, we observed that specimens with strengths below approximately 500 MPa presented a glossy surface, contrasting with the darker, less reflective surfaces of higher-strength specimens, as shown in Figure 8. Each group of specimens consisted of two tensile test specimens. Therefore, two specimens with corresponding numbers were extracted from Group G2A-V. It is evident that there are significant differences in the tensile strength and noticeable variations in surface glossiness among these specimens. This suggests that the DB process applied to the sheet metal may result in varying bond strengths across different regions, possibly due to differences in surface cleanliness or other factors. Further research indicates that minor variations during surface treatment processes could have a significant impact on the mechanical properties of materials. For instance, factors such as surface roughness, the presence of contaminants, and the uniformity of coatings may influence the bond strength. Additionally, factors related to the heat treatment process, such as temperature control, duration, and the cooling rate, could also affect the strength and surface characteristics of materials. These findings emphasize the importance of attention to detail in the manufacturing process to ensure product quality.

**Figure 9:**
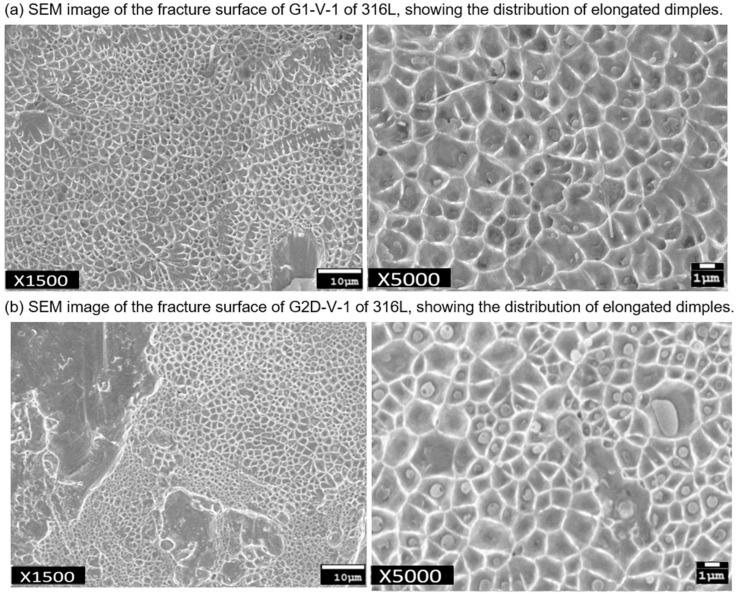
SEM micrographs showing the effect on the topography of fracture surfaces. (**a**) The specimen of G1-V-1 of SS316L. (**b**) The specimen of G2D-V-1 of SS316L.

The microscopic examination of fracture surfaces provides a wealth of information regarding the material’s failure mechanisms. In a brittle fracture, there is a notable absence of plastic deformation, whereas ductile materials, like steel, exhibit extensive plastic deformation, often resulting in “necking” prior to failure, leaving rough fracture surfaces. This distinction is evident upon closer inspection: brittle cracks are characterized by atomically sharp features, progressing bond by bond, whereas ductile fractures display roughness at the microscale, attributed to the formation and coalescence of microscopic voids. To validate this observation, the fracture surfaces of diffusion-bonded SS316L specimens were scrutinized using scanning electron microscopy (SEM). This effect is distinctly discernible in SEM micrographs. As shown in Figure 9, the tensile fracture surface of SS316L exhibits typical dimpling characteristics of ductile materials. 

## 4. Discussion

### 4.1. Tensile Strength, Deformation, and Microstructure Analysis

The tensile tests demonstrated that the deformation behavior varies based on the orientation of the bond with respect to the tensile direction. In particular, the H- and S-type specimens, which had DB joints parallel to the tensile direction, exhibited necking and deformation similar to monolithic materials. This indicates that multi-layered joints in these directions achieve a bonding quality comparable to the parent material, effectively validating the integrity and reliability of the DB process for practical applications. Conversely, the V-type specimens, with DB joints perpendicular to the tensile direction, showed a reduced tensile strength and minimal necking, suggesting a weaker bond in this orientation. This highlights a key consideration for the application of DB in structural components: the orientation of the joint relative to the applied load significantly impacts the mechanical performance.

### 4.2. Specific Product Application and Economic Contributions

Considering the tensile strengths of the SS316L V-type, S-type, and H-type specimens, conditions G1 and G2C exhibited superior tensile strength. Condition G1 demonstrates that with appropriate bonding parameters, the experimental setup can achieve a tensile strength comparable to the parent material, even when the bonding area is increased. Meanwhile, condition G2C shows that increasing the bonding temperature and pressure can significantly shorten the bonding time, thereby enhancing production efficiency. Our results differ from previous DB of stainless steels by demonstrating the effectiveness of multi-layer stacking. The detailed microstructural analysis confirms the formation of robust diffusion bonds with minimal defects, offering a clear advancement in bonding technology. The optimized DB parameters (G2C) identified in our study are particularly suitable for producing SS316L stainless steel cores in compact heat exchangers. These components require high strength and thermal stability, which our bonding process can deliver. By ensuring a superior bond quality, our method can enhance the performance and durability of heat exchangers, making them more reliable for industrial applications. The refined DB process offers potential cost savings by reducing the processing times and energy consumption. The ability to achieve high-quality bonds at optimized temperatures and pressures translates to lower production costs and improved efficiency. Additionally, the enhanced mechanical properties of the bonded joints can lead to a longer service life and reduced maintenance, further contributing to economic benefits.

## 5. Conclusions

The current study predominantly focuses on discussing DB of multi-layered stainless steel. However, a notable imperative remains for the continued refinement of the diffusion welding process. This necessitates the further elucidation of process parameters and the meticulous identification of controls. Additionally, the comprehensive mechanical testing of the diffusion-bonded joints is indispensable to effectively optimize critical parameters such as the temperature, applied pressure, and hold time. Pursuing these endeavors enhances the efficacy of DB techniques and augments the overall understanding and advancement of materials joining methodologies. Therefore, it is imperative to embark upon a rigorous exploration that delves deeper into these intricacies, fostering innovation and progress within the realm of material engineering. The conclusions drawn from this study are summarized as follows:

1. The compression creep rate of SS430 is much higher than that of SS316L.
2. The experimental results of SS316L and SS430’s mechanical properties show that the compressibility of diffusion welding materials does not affect the DB strength.
3. Multi-axial (vertical, horizontal, and diagonal) DB joint tensile strength tests were conducted, indicating that after inspecting the weld bead in different axes, the bonding joint strength was confirmed to be good.
4. The tensile strengths of the monolithic and DB specimens tested in parallel directions are essentially identical, as logically anticipated.
5. The optimized DB parameters (G2C) identified in our study are particularly suitable for producing SS316L stainless steel cores in compact heat exchangers. These parameters provide the best bonding quality and lower cost.

## Figures and Tables

**Table 1:** Chemical composition of SS316L and SS430 stainless steel (wt%).

**Alloy Type**	**Iron**	**Cr**	**Ni**	**Mo**	**Mn**	**Si**	**P**	**N**	**C**	**S**
SS316L	Bal	17	12	2.5	2	0.75	0.05	0.1	0.03	0.03
**Alloy Type**	**Iron**	**Cr**	**Mn**	**Si**	**Ni**	**C**	**P**	**S**		
SS430	Bal	17	1	1	0.75	0.12	0.04	0.03		

**Table 2:** Reported parameters for similar diffusion bonding of 316.

Alloy	Interlayer	Temperature (°C)	Time (min)	Pressure (MPa)	Vacuum	Tensile Strength (MPa)	Ref.
316H	SuperDux 65	1027	30	4.2–7	10^−4^ torr	629~636	[18]
316L	NiP/NiNP	1000	120	10	10^−5^ torr		[19]
316L	-	1100	180	10	10^−5^ torr	566	[12]
316L		945–1100	32–179	7.5–10	10^−6^ torr	350–550	[13]

**Table 3:** Processing parameters for groups G1 and G2 of SS316L and SS430.

Alloy	SS316L					SS430
Processing Parameters	G1	G2A	G2B	G2C	G2D	
Stacking Dim. (mm)	100 × 50 × 3 × 12 pcs	55 × 55 × 3 × 12 pcs				
Vacuum (Torr)	5 × 10^−4^					
Temperature (°C)	1048	1000	1048		1000	1048
Pressure (MPa)	25.3		20.0	25.3	10.0	25.3
Time (min)	25			15	60	30
After DB total Thickness (mm)	35.00	35.10	34.96	34.76	35.30	20.10
Deformation/pcs (mm)	−0.083	−0.075	−0.087	−0.103	−0.058	−1.325
DB compression ratio	2.8%	2.5%	2.9%	3.4%	1.9%	44.2%

**Table 4:** Ultimate tensile strength results at room temperature for parent groups G1 and G2 of SS316L.

SS316L							
Sample No.	Elongation (mm)	Elongation (%)	Cross-Section Area A_0_ (×10^−5^ m^2^)	Max Load (kN)	UTS (MPa)	UTS Avg. (MPa)	DB Ratio (%)
Parent-1	15.20	43.43	3	17.4	578.5	581.7	-
Parent-2	15.24	43.54	3	17.5	582.8		
Parent-3	15.27	43.63	3	17.5	583.9		
G1-V-1	12.30	35.14	3	18.0	599.1	599.7	103.1
G1-V-2	13.87	39.63	3	18.0	600.6		
G2A-V-1	5.71	16.27	3	15.6	518.4	538.0	92.5
G2A-V-2	8.46	24.10	3	16.7	557.6		
G2B-V-1	11.37	32.52	3	15.9	531.5	529.6	91.0
G2B-V-2	11.05	31.61	3	15.8	527.6		
G2C-V-1	8.94	25.72	3	16.9	563.9	576.1	99.0
G2C-V-2	12.88	37.05	3	17.6	588.3		
G2D-V-1	2.72	7.71	3	12.2	407.9	449.8	77.3
G2D-V-2	5.84	16.54	3	14.7	491.6		
G1-S-1	15.38	43.94	3.6	21.1	587.3	589.2	101.3
G1-S-2	15.63	44.66	3.6	21.3	591.2		
G2A-S-1	15.18	43.37	3.6	20.2	560.4	571.0	98.2
G2A-S-2	15.75	45.00	3.6	20.9	581.7		
G2B-S-1	16.25	46.43	3.6	20.5	570.7	579.0	99.5
G2B-S-2	16.12	46.06	3.6	21.1	587.2		
G2C-S-1	16.25	46.43	3.6	19.6	543.4	562.0	96.6
G2C-S-2	16.55	47.29	3.6	20.9	580.5		
G2D-S-1	16.08	45.94	3.6	20.7	574.1	580.9	99.9
G2D-S-2	15.96	45.60	3.6	21.2	587.8		
G1-H-1	16.02	45.77	3	17.6	586.5	592.7	101.9
G1-H-2	15.46	44.17	3	18.0	598.9		
G2A-H-1	15.33	43.80	3	18.1	604.3	602.3	103.5
G2A-H-2	15.99	45.69	3	18.0	600.2		
G2B-H-1	16.44	46.97	3	17.8	591.9	580.2	99.7
G2B-H-2	16.19	46.26	3	17.1	568.4		
G2C-H-1	15.09	43.11	3	17.1	570.7	575.0	98.8
G2C-H-2	16.13	46.09	3	17.4	579.3		
G2D-H-1	16.16	46.17	3	17.2	572.9	572.3	98.4
G2D-H-2	15.93	45.51	3	17.2	571.8		

**Table 5:** Ultimate tensile strength results at room temperature for parent and specimens of SS430.

430							
Sample No.	Elongation (mm)	Elongation (%)	Cross-Section Area A_0_ (×10^−5^ m^2^)	Max Load (kN)	UTS (MPa)	UTS Avg. (MPa)	DB Ratio (%)
Parent-1	6.09	17.40	3	12.8	426.1	425.9	-
Parent-2	6.12	17.49	3	12.9	431.1		
Parent-3	5.64	16.11	3	12.6	420.7		
V-1	0.84	4.20	3	18.4	614.4	533.6	125.3%
V-2	0.86	4.30	3	17.7	589.1		
V-3	0.81	4.05	3	13.1	435.7		
V-4	0.87	4.35	3	14.7	490.7		
V-5	1.04	5.20	3	15.3	509.4		
V-6	0.95	4.75	3	16.9	562.4		
H-1	2.77	13.85	3	16.9	562.9	578.6	135.8
H-2	2.50	12.50	3	17.0	565.5		
H-3	2.22	11.10	3	17.4	579.8		
H-4	2.73	13.65	3	17.4	581.2		
H-5	2.32	11.60	3	17.3	576.0		
H-6	2.67	13.35	3	18.2	605.9		

## Data Availability

Data will be made available on request.
